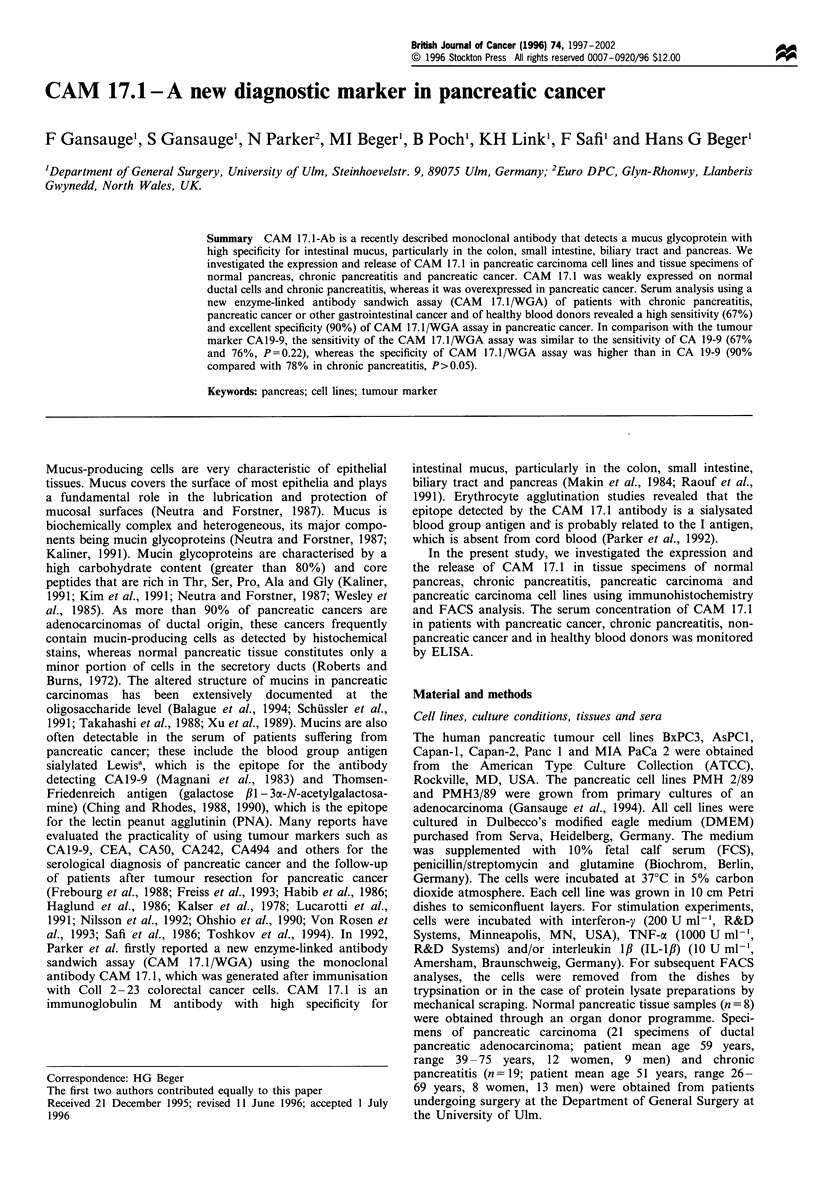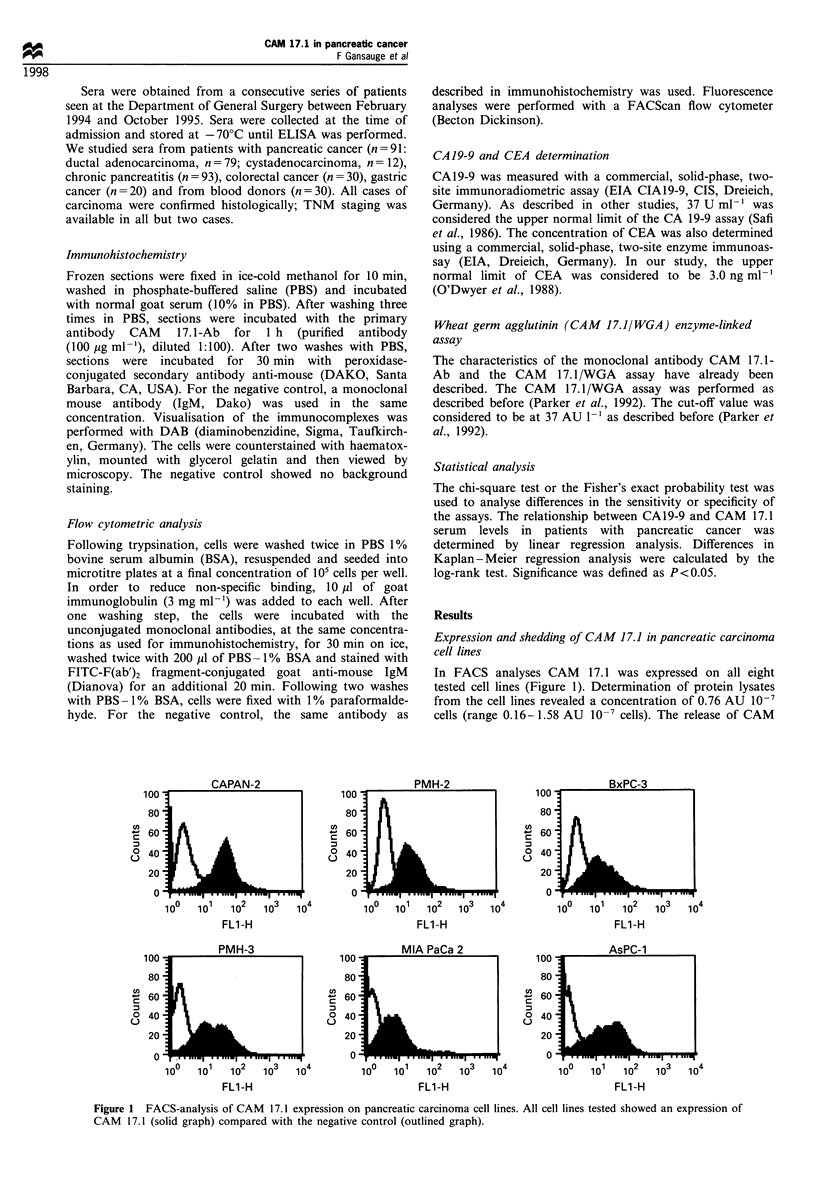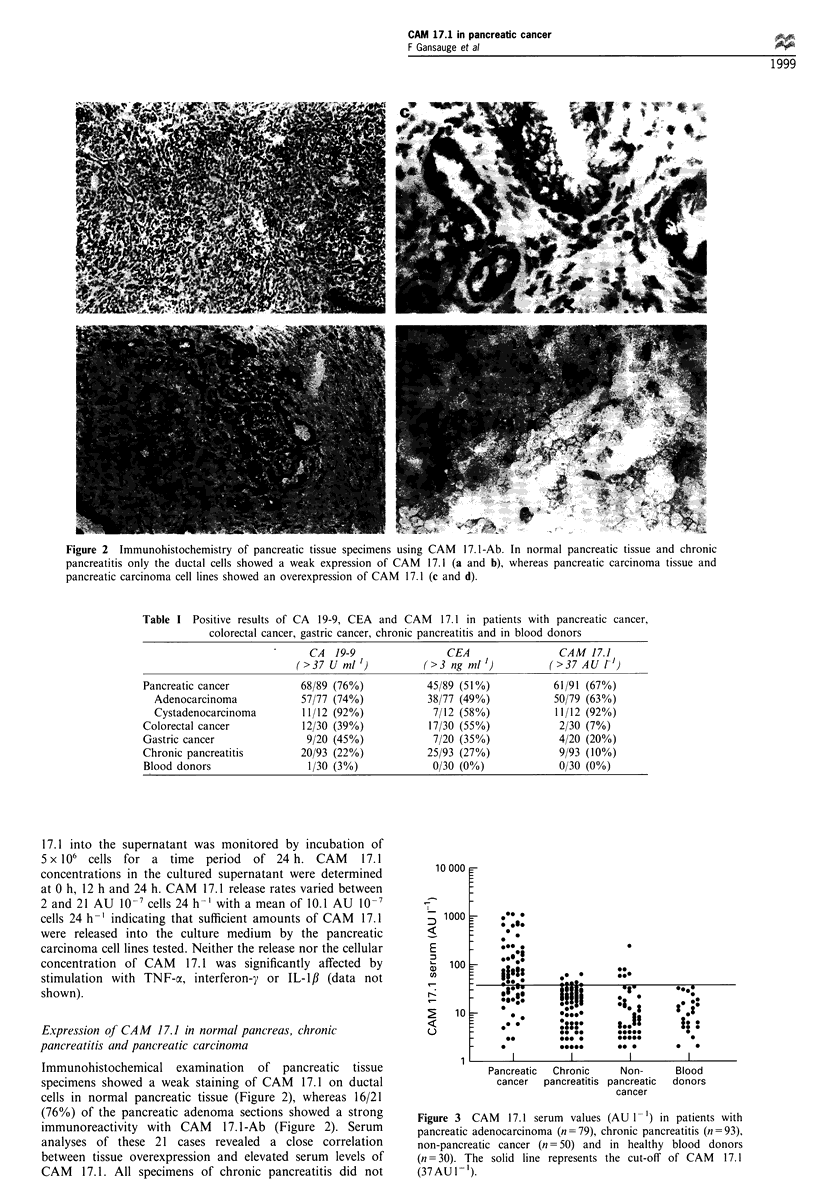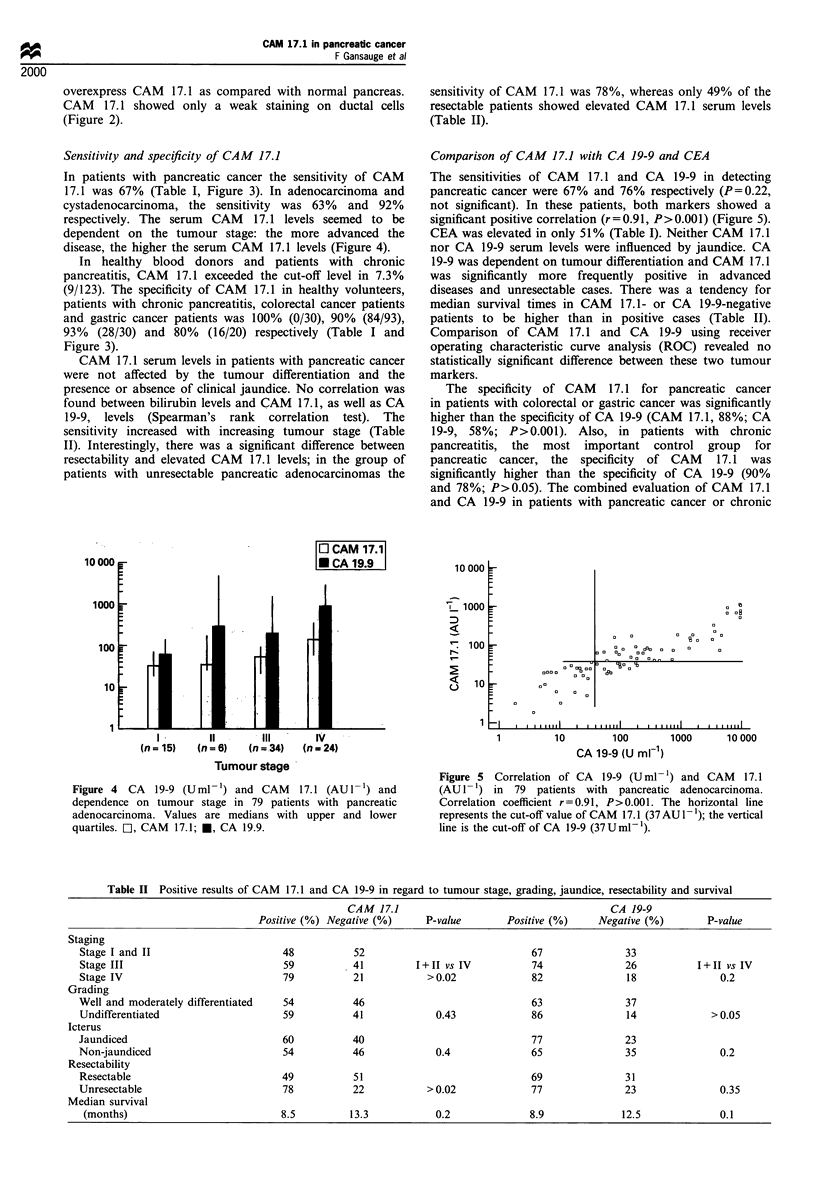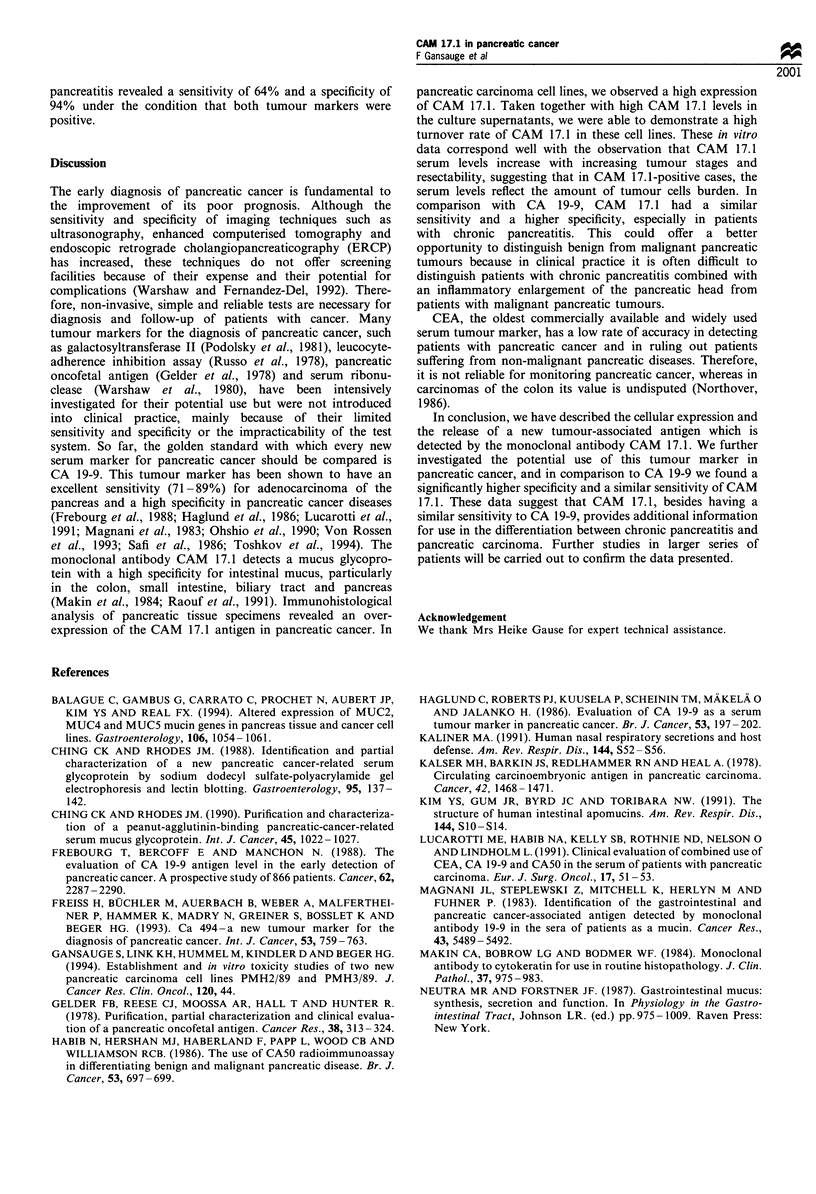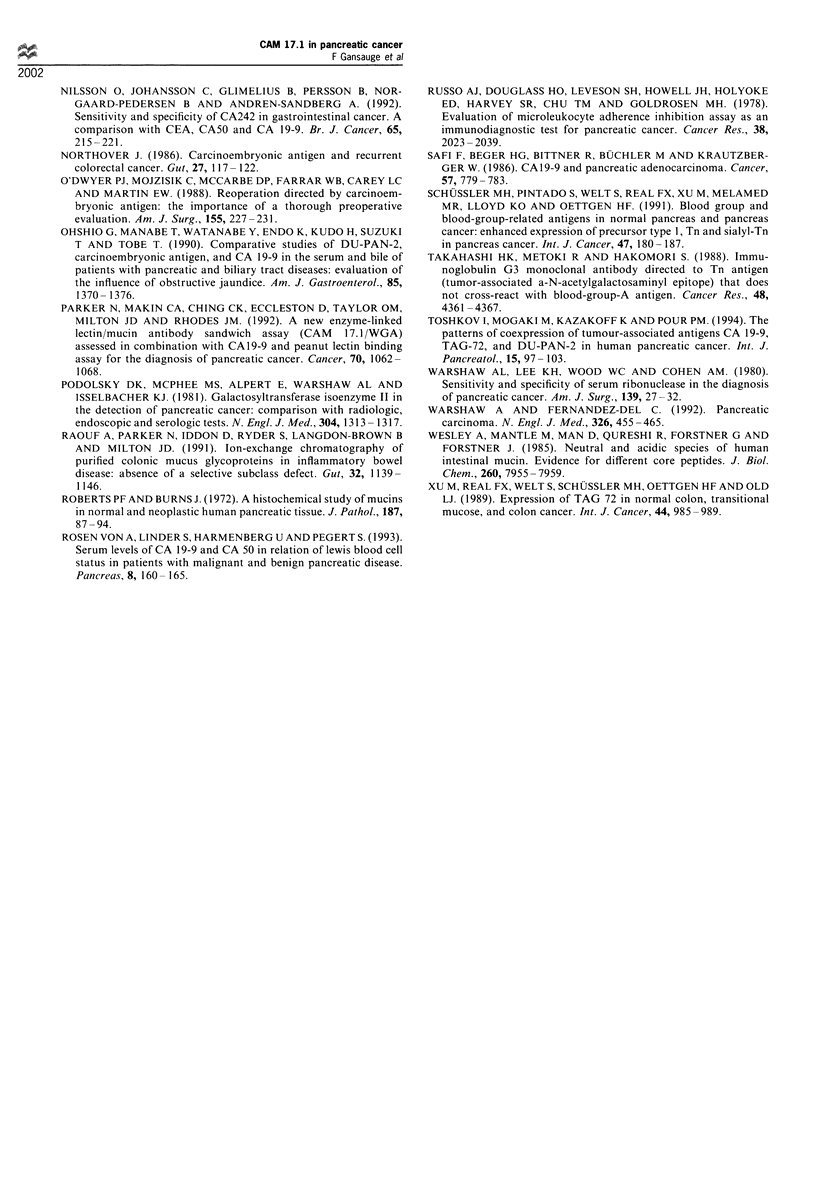# CAM 17.1--a new diagnostic marker in pancreatic cancer.

**DOI:** 10.1038/bjc.1996.666

**Published:** 1996-12

**Authors:** F. Gansauge, S. Gansauge, N. Parker, M. I. Beger, B. Poch, K. H. Link, F. Safi, H. G. Beger

**Affiliations:** Department of General Surgery, University of Ulm, Germany.

## Abstract

**Images:**


British Journal of Cancer (1996) 74, 1997-2002
© 1996 Stockton Press All rights reserved 0007-0920/96 $12.00

### CAM 17.1 - A new diagnostic marker in pancreatic cancer

F Gansauge[1], S Gansauge[1], N Parker[2], MI Beger[1], B Poch[1], KH Link[1], F Safi[1] and Hans G Beger[1]

[1]*Department of General Surgery, University of Ulm, Steinhoevelstr. 9, 89075 Ulm, Germany;* [2]*Euro DPC, Glyn-Rhonwy, Llanberis Gwynedd, North Wales, UK.*

**Summary** CAM 17.1-Ab is a recently described monoclonal antibody that detects a mucus glycoprotein with high specificity for intestinal mucus, particularly in the colon, small intestine, biliary tract and pancreas. We investigated the expression and release of CAM 17.1 in pancreatic carcinoma cell lines and tissue specimens of normal pancreas, chronic pancreatitis and pancreatic cancer. CAM 17.1 was weakly expressed on normal ductal cells and chronic pancreatitis, whereas it was overexpressed in pancreatic cancer. Serum analysis using a new enzyme-linked antibody sandwich assay (CAM 17.1/WGA) of patients with chronic pancreatitis, pancreatic cancer or other gastrointestinal cancer and of healthy blood donors revealed a high sensitivity (67%) and excellent specificity (90%) of CAM 17.1/WGA assay in pancreatic cancer. In comparison with the tumour marker CA19-9, the sensitivity of the CAM 17.1/WGA assay was similar to the sensitivity of CA 19-9 (67% and 76%, $P=0.22$), whereas the specificity of CAM 17.1/WGA assay was higher than in CA 19-9 (90% compared with 78% in chronic pancreatitis, $P>0.05$).

**Keywords:** pancreas; cell lines; tumour marker

Mucus-producing cells are very characteristic of epithelial tissues. Mucus covers the surface of most epithelia and plays a fundamental role in the lubrication and protection of mucosal surfaces (Neutra and Forstner, 1987). Mucus is biochemically complex and heterogeneous, its major components being mucin glycoproteins (Neutra and Forstner, 1987; Kaliner, 1991). Mucin glycoproteins are characterised by a high carbohydrate content (greater than 80%) and core peptides that are rich in Thr, Ser, Pro, Ala and Gly (Kaliner, 1991; Kim *et al.*, 1991; Neutra and Forstner, 1987; Wesley *et al.*, 1985). As more than 90% of pancreatic cancers are adenocarcinomas of ductal origin, these cancers frequently contain mucin-producing cells as detected by histochemical stains, whereas normal pancreatic tissue constitutes only a minor portion of cells in the secretory ducts (Roberts and Burns, 1972). The altered structure of mucins in pancreatic carcinomas has been extensively documented at the oligosaccharide level (Balague *et al.*, 1994; Schüssler *et al.*, 1991; Takahashi *et al.*, 1988; Xu *et al.*, 1989). Mucins are also often detectable in the serum of patients suffering from pancreatic cancer; these include the blood group antigen sialylated Lewis[a], which is the epitope for the antibody detecting CA19-9 (Magnani *et al.*, 1983) and Thomsen-Friedenreich antigen (galactose $\beta 1-3\alpha$-*N*-acetylgalactosamine) (Ching and Rhodes, 1988, 1990), which is the epitope for the lectin peanut agglutinin (PNA). Many reports have evaluated the practicality of using tumour markers such as CA19-9, CEA, CA50, CA242, CA494 and others for the serological diagnosis of pancreatic cancer and the follow-up of patients after tumour resection for pancreatic cancer (Frebourg *et al.*, 1988; Freiss *et al.*, 1993; Habib *et al.*, 1986; Haglund *et al.*, 1986; Kalser *et al.*, 1978; Lucarotti *et al.*, 1991; Nilsson *et al.*, 1992; Ohshio *et al.*, 1990; Von Rosen *et al.*, 1993; Safi *et al.*, 1986; Toshkov *et al.*, 1994). In 1992, Parker *et al.* firstly reported a new enzyme-linked antibody sandwich assay (CAM 17.1/WGA) using the monoclonal antibody CAM 17.1, which was generated after immunisation with Coll 2-23 colorectal cancer cells. CAM 17.1 is an immunoglobulin M antibody with high specificity for intestinal mucus, particularly in the colon, small intestine, biliary tract and pancreas (Makin *et al.*, 1984; Raouf *et al.*, 1991). Erythrocyte agglutination studies revealed that the epitope detected by the CAM 17.1 antibody is a sialysated blood group antigen and is probably related to the I antigen, which is absent from cord blood (Parker *et al.*, 1992).

In the present study, we investigated the expression and the release of CAM 17.1 in tissue specimens of normal pancreas, chronic pancreatitis, pancreatic carcinoma and pancreatic carcinoma cell lines using immunohistochemistry and FACS analysis. The serum concentration of CAM 17.1 in patients with pancreatic cancer, chronic pancreatitis, non-pancreatic cancer and in healthy blood donors was monitored by ELISA.

### Material and methods

### *Cell lines, culture conditions, tissues and sera*

The human pancreatic tumour cell lines BxPC3, AsPCl, Capan-1, Capan-2, Panc 1 and MIA PaCa 2 were obtained from the American Type Culture Collection (ATCC), Rockville, MD, USA. The pancreatic cell lines PMH 2/89 and PMH3/89 were grown from primary cultures of an adenocarcinoma (Gansauge *et al.*, 1994). All cell lines were cultured in Dulbecco's modified eagle medium (DMEM) purchased from Serva, Heidelberg, Germany. The medium was supplemented with 10% fetal calf serum (FCS), penicillin/streptomycin and glutamine (Biochrom, Berlin, Germany). The cells were incubated at 37°C in 5% carbon dioxide atmosphere. Each cell line was grown in 10 cm Petri dishes to semiconfluent layers. For stimulation experiments, cells were incubated with interferon-$\gamma$ (200 U $ml^{-1}$, R&D Systems, Minneapolis, MN, USA), TNF-$\alpha$ (1000 U $ml^{-1}$, R&D Systems) and/or interleukin $1\beta$ (IL-$1\beta$) (10 U $ml^{-1}$, Amersham, Braunschweig, Germany). For subsequent FACS analyses, the cells were removed from the dishes by trypsination or in the case of protein lysate preparations by mechanical scraping. Normal pancreatic tissue samples ($n=8$) were obtained through an organ donor programme. Specimens of pancreatic carcinoma (21 specimens of ductal pancreatic adenocarcinoma; patient mean age 59 years, range 39-75 years, 12 women, 9 men) and chronic pancreatitis ($n=19$; patient mean age 51 years, range 26-69 years, 8 women, 13 men) were obtained from patients undergoing surgery at the Department of General Surgery at the University of Ulm.

Correspondence: HG Beger
The first two authors contributed equally to this paper
Received 21 December 1995; revised 11 June 1996; accepted 1 July 1996

Sera were obtained from a consecutive series of patients seen at the Department of General Surgery between February 1994 and October 1995. Sera were collected at the time of admission and stored at −70°C until ELISA was performed. We studied sera from patients with pancreatic cancer ($n=91$: ductal adenocarcinoma, $n=79$; cystadenocarcinoma, $n=12$), chronic pancreatitis ($n=93$), colorectal cancer ($n=30$), gastric cancer ($n=20$) and from blood donors ($n=30$). All cases of carcinoma were confirmed histologically; TNM staging was available in all but two cases.

*Immunohistochemistry*

Frozen sections were fixed in ice-cold methanol for 10 min, washed in phosphate-buffered saline (PBS) and incubated with normal goat serum (10% in PBS). After washing three times in PBS, sections were incubated with the primary antibody CAM 17.1-Ab for 1 h (purified antibody (100 $\mu$g ml$^{-1}$), diluted 1:100). After two washes with PBS, sections were incubated for 30 min with peroxidase-conjugated secondary antibody anti-mouse (DAKO, Santa Barbara, CA, USA). For the negative control, a monoclonal mouse antibody (IgM, Dako) was used in the same concentration. Visualisation of the immunocomplexes was performed with DAB (diaminobenzidine, Sigma, Taufkirchen, Germany). The cells were counterstained with haematoxylin, mounted with glycerol gelatin and then viewed by microscopy. The negative control showed no background staining.

*Flow cytometric analysis*

Following trypsination, cells were washed twice in PBS 1% bovine serum albumin (BSA), resuspended and seeded into microtitre plates at a final concentration of $10^5$ cells per well. In order to reduce non-specific binding, 10 $\mu$l of goat immunoglobulin (3 mg ml$^{-1}$) was added to each well. After one washing step, the cells were incubated with the unconjugated monoclonal antibodies, at the same concentrations as used for immunohistochemistry, for 30 min on ice, washed twice with 200 $\mu$l of PBS−1% BSA and stained with FITC-F(ab')$_2$ fragment-conjugated goat anti-mouse IgM (Dianova) for an additional 20 min. Following two washes with PBS−1% BSA, cells were fixed with 1% paraformaldehyde. For the negative control, the same antibody as described in immunohistochemistry was used. Fluorescence analyses were performed with a FACScan flow cytometer (Becton Dickinson).

*CA19-9 and CEA determination*

CA19-9 was measured with a commercial, solid-phase, two-site immunoradiometric assay (EIA CIA19-9, CIS, Dreieich, Germany). As described in other studies, 37 U ml$^{-1}$ was considered the upper normal limit of the CA 19-9 assay (Safi *et al.*, 1986). The concentration of CEA was also determined using a commercial, solid-phase, two-site enzyme immunoassay (EIA, Dreieich, Germany). In our study, the upper normal limit of CEA was considered to be 3.0 ng ml$^{-1}$ (O'Dwyer *et al.*, 1988).

*Wheat germ agglutinin (CAM 17.1/WGA) enzyme-linked assay*

The characteristics of the monoclonal antibody CAM 17.1-Ab and the CAM 17.1/WGA assay have already been described. The CAM 17.1/WGA assay was performed as described before (Parker *et al.*, 1992). The cut-off value was considered to be at 37 AU l$^{-1}$ as described before (Parker *et al.*, 1992).

*Statistical analysis*

The chi-square test or the Fisher's exact probability test was used to analyse differences in the sensitivity or specificity of the assays. The relationship between CA19-9 and CAM 17.1 serum levels in patients with pancreatic cancer was determined by linear regression analysis. Differences in Kaplan−Meier regression analysis were calculated by the log-rank test. Significance was defined as $P<0.05$.

### Results

*Expression and shedding of CAM 17.1 in pancreatic carcinoma cell lines*

In FACS analyses CAM 17.1 was expressed on all eight tested cell lines (Figure 1). Determination of protein lysates from the cell lines revealed a concentration of 0.76 AU $10^{-7}$ cells (range 0.16−1.58 AU $10^{-7}$ cells). The release of CAM

**Figure 1** FACS-analysis of CAM 17.1 expression on pancreatic carcinoma cell lines. All cell lines tested showed an expression of CAM 17.1 (solid graph) compared with the negative control (outlined graph).

**Figure 2** Immunohistochemistry of pancreatic tissue specimens using CAM 17.1-Ab. In normal pancreatic tissue and chronic pancreatitis only the ductal cells showed a weak expression of CAM 17.1 (**a** and **b**), whereas pancreatic carcinoma tissue and pancreatic carcinoma cell lines showed an overexpression of CAM 17.1 (**c** and **d**).

**Table I** Positive results of CA 19-9, CEA and CAM 17.1 in patients with pancreatic cancer, colorectal cancer, gastric cancer, chronic pancreatitis and in blood donors

| | *CA 19-9 (>37 U ml$^{-1}$)* | *CEA (>3 ng ml$^{-1}$)* | *CAM 17.1 (>37 AU l$^{-1}$)* |
|---|---|---|---|
| Pancreatic cancer | 68/89 (76%) | 45/89 (51%) | 61/91 (67%) |
| Adenocarcinoma | 57/77 (74%) | 38/77 (49%) | 50/79 (63%) |
| Cystadenocarcinoma | 11/12 (92%) | 7/12 (58%) | 11/12 (92%) |
| Colorectal cancer | 12/30 (39%) | 17/30 (55%) | 2/30 (7%) |
| Gastric cancer | 9/20 (45%) | 7/20 (35%) | 4/20 (20%) |
| Chronic pancreatitis | 20/93 (22%) | 25/93 (27%) | 9/93 (10%) |
| Blood donors | 1/30 (3%) | 0/30 (0%) | 0/30 (0%) |

17.1 into the supernatant was monitored by incubation of $5 \times 10^6$ cells for a time period of 24 h. CAM 17.1 concentrations in the cultured supernatant were determined at 0 h, 12 h and 24 h. CAM 17.1 release rates varied between 2 and 21 AU $10^{-7}$ cells 24 h$^{-1}$ with a mean of 10.1 AU $10^{-7}$ cells 24 h$^{-1}$ indicating that sufficient amounts of CAM 17.1 were released into the culture medium by the pancreatic carcinoma cell lines tested. Neither the release nor the cellular concentration of CAM 17.1 was significantly affected by stimulation with TNF-$\alpha$, interferon-$\gamma$ or IL-1$\beta$ (data not shown).

### *Expression of CAM 17.1 in normal pancreas, chronic pancreatitis and pancreatic carcinoma*

Immunohistochemical examination of pancreatic tissue specimens showed a weak staining of CAM 17.1 on ductal cells in normal pancreatic tissue (Figure 2), whereas 16/21 (76%) of the pancreatic adenoma sections showed a strong immunoreactivity with CAM 17.1-Ab (Figure 2). Serum analyses of these 21 cases revealed a close correlation between tissue overexpression and elevated serum levels of CAM 17.1. All specimens of chronic pancreatitis did not

**Figure 3** CAM 17.1 serum values (AU l$^{-1}$) in patients with pancreatic adenocarcinoma ($n=79$), chronic pancreatitis ($n=93$), non-pancreatic cancer ($n=50$) and in healthy blood donors ($n=30$). The solid line represents the cut-off of CAM 17.1 (37 AU l$^{-1}$).

overexpress CAM 17.1 as compared with normal pancreas. CAM 17.1 showed only a weak staining on ductal cells (Figure 2).

*Sensitivity and specificity of CAM 17.1*

In patients with pancreatic cancer the sensitivity of CAM 17.1 was 67% (Table I, Figure 3). In adenocarcinoma and cystadenocarcinoma, the sensitivity was 63% and 92% respectively. The serum CAM 17.1 levels seemed to be dependent on the tumour stage: the more advanced the disease, the higher the serum CAM 17.1 levels (Figure 4).

In healthy blood donors and patients with chronic pancreatitis, CAM 17.1 exceeded the cut-off level in 7.3% (9/123). The specificity of CAM 17.1 in healthy volunteers, patients with chronic pancreatitis, colorectal cancer patients and gastric cancer patients was 100% (0/30), 90% (84/93), 93% (28/30) and 80% (16/20) respectively (Table I and Figure 3).

CAM 17.1 serum levels in patients with pancreatic cancer were not affected by the tumour differentiation and the presence or absence of clinical jaundice. No correlation was found between bilirubin levels and CAM 17.1, as well as CA 19-9, levels (Spearman's rank correlation test). The sensitivity increased with increasing tumour stage (Table II). Interestingly, there was a significant difference between resectability and elevated CAM 17.1 levels; in the group of patients with unresectable pancreatic adenocarcinomas the sensitivity of CAM 17.1 was 78%, whereas only 49% of the resectable patients showed elevated CAM 17.1 serum levels (Table II).

*Comparison of CAM 17.1 with CA 19-9 and CEA*

The sensitivities of CAM 17.1 and CA 19-9 in detecting pancreatic cancer were 67% and 76% respectively ($P=0.22$, not significant). In these patients, both markers showed a significant positive correlation ($r=0.91$, $P>0.001$) (Figure 5). CEA was elevated in only 51% (Table I). Neither CAM 17.1 nor CA 19-9 serum levels were influenced by jaundice. CA 19-9 was dependent on tumour differentiation and CAM 17.1 was significantly more frequently positive in advanced diseases and unresectable cases. There was a tendency for median survival times in CAM 17.1- or CA 19-9-negative patients to be higher than in positive cases (Table II). Comparison of CAM 17.1 and CA 19-9 using receiver operating characteristic curve analysis (ROC) revealed no statistically significant difference between these two tumour markers.

The specificity of CAM 17.1 for pancreatic cancer in patients with colorectal or gastric cancer was significantly higher than the specificity of CA 19-9 (CAM 17.1, 88%; CA 19-9, 58%; $P>0.001$). Also, in patients with chronic pancreatitis, the most important control group for pancreatic cancer, the specificity of CAM 17.1 was significantly higher than the specificity of CA 19-9 (90% and 78%; $P>0.05$). The combined evaluation of CAM 17.1 and CA 19-9 in patients with pancreatic cancer or chronic

**Figure 4** CA 19-9 ($U ml^{-1}$) and CAM 17.1 ($AU l^{-1}$) and dependence on tumour stage in 79 patients with pancreatic adenocarcinoma. Values are medians with upper and lower quartiles. □, CAM 17.1; ■, CA 19.9.

**Figure 5** Correlation of CA 19-9 ($U ml^{-1}$) and CAM 17.1 ($AU l^{-1}$) in 79 patients with pancreatic adenocarcinoma. Correlation coefficient $r=0.91$, $P>0.001$. The horizontal line represents the cut-off value of CAM 17.1 ($37 AU l^{-1}$); the vertical line is the cut-off of CA 19-9 ($37 U ml^{-1}$).

**Table II** Positive results of CAM 17.1 and CA 19-9 in regard to tumour stage, grading, jaundice, resectability and survival

| | *CAM 17.1* | | | *CA 19-9* | | |
|---|---|---|---|---|---|---|
| | *Positive* (%) | *Negative* (%) | P-*value* | *Positive* (%) | *Negative* (%) | P-*value* |
| Staging | | | | | | |
| Stage I and II | 48 | 52 | | 67 | 33 | |
| Stage III | 59 | 41 | I+II *vs* IV | 74 | 26 | I+II *vs* IV |
| Stage IV | 79 | 21 | >0.02 | 82 | 18 | 0.2 |
| Grading | | | | | | |
| Well and moderately differentiated | 54 | 46 | | 63 | 37 | |
| Undifferentiated | 59 | 41 | 0.43 | 86 | 14 | >0.05 |
| Icterus | | | | | | |
| Jaundiced | 60 | 40 | | 77 | 23 | |
| Non-jaundiced | 54 | 46 | 0.4 | 65 | 35 | 0.2 |
| Resectability | | | | | | |
| Resectable | 49 | 51 | | 69 | 31 | |
| Unresectable | 78 | 22 | >0.02 | 77 | 23 | 0.35 |
| Median survival (months) | 8.5 | 13.3 | 0.2 | 8.9 | 12.5 | 0.1 |

pancreatitis revealed a sensitivity of 64% and a specificity of 94% under the condition that both tumour markers were positive.

### Discussion

The early diagnosis of pancreatic cancer is fundamental to the improvement of its poor prognosis. Although the sensitivity and specificity of imaging techniques such as ultrasonography, enhanced computerised tomography and endoscopic retrograde cholangiopancreaticography (ERCP) has increased, these techniques do not offer screening facilities because of their expense and their potential for complications (Warshaw and Fernandez-Del, 1992). Therefore, non-invasive, simple and reliable tests are necessary for diagnosis and follow-up of patients with cancer. Many tumour markers for the diagnosis of pancreatic cancer, such as galactosyltransferase II (Podolsky *et al.*, 1981), leucocyte-adherence inhibition assay (Russo *et al.*, 1978), pancreatic oncofetal antigen (Gelder *et al.*, 1978) and serum ribonuclease (Warshaw *et al.*, 1980), have been intensively investigated for their potential use but were not introduced into clinical practice, mainly because of their limited sensitivity and specificity or the impracticability of the test system. So far, the golden standard with which every new serum marker for pancreatic cancer should be compared is CA 19-9. This tumour marker has been shown to have an excellent sensitivity (71–89%) for adenocarcinoma of the pancreas and a high specificity in pancreatic cancer diseases (Frebourg *et al.*, 1988; Haglund *et al.*, 1986; Lucarotti *et al.*, 1991; Magnani *et al.*, 1983; Ohshio *et al.*, 1990; Von Rossen *et al.*, 1993; Safi *et al.*, 1986; Toshkov *et al.*, 1994). The monoclonal antibody CAM 17.1 detects a mucus glycoprotein with a high specificity for intestinal mucus, particularly in the colon, small intestine, biliary tract and pancreas (Makin *et al.*, 1984; Raouf *et al.*, 1991). Immunohistological analysis of pancreatic tissue specimens revealed an overexpression of the CAM 17.1 antigen in pancreatic cancer. In pancreatic carcinoma cell lines, we observed a high expression of CAM 17.1. Taken together with high CAM 17.1 levels in the culture supernatants, we were able to demonstrate a high turnover rate of CAM 17.1 in these cell lines. These *in vitro* data correspond well with the observation that CAM 17.1 serum levels increase with increasing tumour stages and resectability, suggesting that in CAM 17.1-positive cases, the serum levels reflect the amount of tumour cells burden. In comparison with CA 19-9, CAM 17.1 had a similar sensitivity and a higher specificity, especially in patients with chronic pancreatitis. This could offer a better opportunity to distinguish benign from malignant pancreatic tumours because in clinical practice it is often difficult to distinguish patients with chronic pancreatitis combined with an inflammatory enlargement of the pancreatic head from patients with malignant pancreatic tumours.

CEA, the oldest commercially available and widely used serum tumour marker, has a low rate of accuracy in detecting patients with pancreatic cancer and in ruling out patients suffering from non-malignant pancreatic diseases. Therefore, it is not reliable for monitoring pancreatic cancer, whereas in carcinomas of the colon its value is undisputed (Northover, 1986).

In conclusion, we have described the cellular expression and the release of a new tumour-associated antigen which is detected by the monoclonal antibody CAM 17.1. We further investigated the potential use of this tumour marker in pancreatic cancer, and in comparison to CA 19-9 we found a significantly higher specificity and a similar sensitivity of CAM 17.1. These data suggest that CAM 17.1, besides having a similar sensitivity to CA 19-9, provides additional information for use in the differentiation between chronic pancreatitis and pancreatic carcinoma. Further studies in larger series of patients will be carried out to confirm the data presented.

**Acknowledgement**
We thank Mrs Heike Gause for expert technical assistance.

### References

BALAGUE C, GAMBUS G, CARRATO C, PROCHET N, AUBERT JP, KIM YS AND REAL FX. (1994). Altered expression of MUC2, MUC4 and MUC5 mucin genes in pancreas tissue and cancer cell lines. *Gastroenterology*, **106**, 1054–1061.

CHING CK AND RHODES JM. (1988). Identification and partial characterization of a new pancreatic cancer-related serum glycoprotein by sodium dodecyl sulfate-polyacrylamide gel electrophoresis and lectin blotting. *Gastroenterology*, **95**, 137–142.

CHING CK AND RHODES JM. (1990). Purification and characterization of a peanut-agglutinin-binding pancreatic-cancer-related serum mucus glycoprotein. *Int. J. Cancer*, **45**, 1022–1027.

FREBOURG T, BERCOFF E AND MANCHON N. (1988). The evaluation of CA 19-9 antigen level in the early detection of pancreatic cancer. A prospective study of 866 patients. *Cancer*, **62**, 2287–2290.

FREISS H, BÜCHLER M, AUERBACH B, WEBER A, MALFERTHEINER P, HAMMER K, MADRY N, GREINER S, BOSSLET K AND BEGER HG. (1993). Ca 494-a new tumour marker for the diagnosis of pancreatic cancer. *Int. J. Cancer*, **53**, 759–763.

GANSAUGE S, LINK KH, HUMMEL M, KINDLER D AND BEGER HG. (1994). Establishment and *in vitro* toxicity studies of two new pancreatic carcinoma cell lines PMH2/89 and PMH3/89. *J. Cancer Res. Clin. Oncol.*, **120**, 44.

GELDER FB, REESE CJ, MOOSSA AR, HALL T AND HUNTER R. (1978). Purification, partial characterization and clinical evaluation of a pancreatic oncofetal antigen. *Cancer Res.*, **38**, 313–324.

HABIB N, HERSHAN MJ, HABERLAND F, PAPP L, WOOD CB AND WILLIAMSON RCB. (1986). The use of CA50 radioimmunoassay in differentiating benign and malignant pancreatic disease. *Br. J. Cancer*, **53**, 697–699.

HAGLUND C, ROBERTS PJ, KUUSELA P, SCHEININ TM, MÄKELÄ O AND JALANKO H. (1986). Evaluation of CA 19-9 as a serum tumour marker in pancreatic cancer. *Br. J. Cancer*, **53**, 197–202.

KALINER MA. (1991). Human nasal respiratory secretions and host defense. *Am. Rev. Respir. Dis.*, **144**, S52–S56.

KALSER MH, BARKIN JS, REDLHAMMER RN AND HEAL A. (1978). Circulating carcinoembryonic antigen in pancreatic carcinoma. *Cancer*, *42*, 1468–1471.

KIM YS, GUM JR, BYRD JC AND TORIBARA NW. (1991). The structure of human intestinal apomucins. *Am. Rev. Respir. Dis.*, **144**, S10–S14.

LUCAROTTI ME, HABIB NA, KELLY SB, ROTHNIE ND, NELSON O AND LINDHOLM L. (1991). Clinical evaluation of combined use of CEA, CA 19-9 and CA50 in the serum of patients with pancreatic carcinoma. *Eur. J. Surg. Oncol.*, **17**, 51–53.

MAGNANI JL, STEPLEWSKI Z, MITCHELL K, HERLYN M AND FUHNER P. (1983). Identification of the gastrointestinal and pancreatic cancer-associated antigen detected by monoclonal antibody 19-9 in the sera of patients as a mucin. *Cancer Res.*, **43**, 5489–5492.

MAKIN CA, BOBROW LG AND BODMER WF. (1984). Monoclonal antibody to cytokeratin for use in routine histopathology. *J. Clin. Pathol.*, **37**, 975–983.

NEUTRA MR AND FORSTNER JF. (1987). Gastrointestinal mucus: synthesis, secretion and function. In *Physiology in the Gastrointestinal Tract*, Johnson LR. (ed.) pp.975–1009. Raven Press: New York.

NILSSON O, JOHANSSON C, GLIMELIUS B, PERSSON B, NORGAARD-PEDERSEN B AND ANDREN-SANDBERG A. (1992). Sensitivity and specificity of CA242 in gastrointestinal cancer. A comparison with CEA, CA50 and CA 19-9. *Br. J. Cancer*, **65,** 215–221.

NORTHOVER J. (1986). Carcinoembryonic antigen and recurrent colorectal cancer. *Gut*, **27,** 117–122.

O'DWYER PJ, MOJZISIK C, MCCARBE DP, FARRAR WB, CAREY LC AND MARTIN EW. (1988). Reoperation directed by carcinoembryonic antigen: the importance of a thorough preoperative evaluation. *Am. J. Surg.*, **155,** 227–231.

OHSHIO G, MANABE T, WATANABE Y, ENDO K, KUDO H, SUZUKI T AND TOBE T. (1990). Comparative studies of DU-PAN-2, carcinoembryonic antigen, and CA 19-9 in the serum and bile of patients with pancreatic and biliary tract diseases: evaluation of the influence of obstructive jaundice. *Am. J. Gastroenterol.*, **85,** 1370–1376.

PARKER N, MAKIN CA, CHING CK, ECCLESTON D, TAYLOR OM, MILTON JD AND RHODES JM. (1992). A new enzyme-linked lectin/mucin antibody sandwich assay (CAM 17.1/WGA) assessed in combination with CA19-9 and peanut lectin binding assay for the diagnosis of pancreatic cancer. *Cancer*, **70,** 1062–1068.

PODOLSKY DK, MCPHEE MS, ALPERT E, WARSHAW AL AND ISSELBACHER KJ. (1981). Galactosyltransferase isoenzyme II in the detection of pancreatic cancer: comparison with radiologic, endoscopic and serologic tests. *N. Engl. J. Med.*, **304,** 1313–1317.

RAOUF A, PARKER N, IDDON D, RYDER S, LANGDON-BROWN B AND MILTON JD. (1991). Ion-exchange chromatography of purified colonic mucus glycoproteins in inflammatory bowel disease: absence of a selective subclass defect. *Gut*, **32,** 1139–1146.

ROBERTS PF AND BURNS J. (1972). A histochemical study of mucins in normal and neoplastic human pancreatic tissue. *J. Pathol.*, **187,** 87–94.

ROSEN VON A, LINDER S, HARMENBERG U AND PEGERT S. (1993). Serum levels of CA 19-9 and CA 50 in relation of lewis blood cell status in patients with malignant and benign pancreatic disease. *Pancreas*, **8,** 160–165.

RUSSO AJ, DOUGLASS HO, LEVESON SH, HOWELL JH, HOLYOKE ED, HARVEY SR, CHU TM AND GOLDROSEN MH. (1978). Evaluation of microleukocyte adherence inhibition assay as an immunodiagnostic test for pancreatic cancer. *Cancer Res.*, **38,** 2023–2039.

SAFI F, BEGER HG, BITTNER R, BÜCHLER M AND KRAUTZBERGER W. (1986). CA19-9 and pancreatic adenocarcinoma. *Cancer*, **57,** 779–783.

SCHÜSSLER MH, PINTADO S, WELT S, REAL FX, XU M, MELAMED MR, LLOYD KO AND OETTGEN HF. (1991). Blood group and blood-group-related antigens in normal pancreas and pancreas cancer: enhanced expression of precursor type 1, Tn and sialyl-Tn in pancreas cancer. *Int. J. Cancer*, **47,** 180–187.

TAKAHASHI HK, METOKI R AND HAKOMORI S. (1988). Immunoglobulin G3 monoclonal antibody directed to Tn antigen (tumor-associated a-N-acetylgalactosaminyl epitope) that does not cross-react with blood-group-A antigen. *Cancer Res.*, **48,** 4361–4367.

TOSHKOV I, MOGAKI M, KAZAKOFF K AND POUR PM. (1994). The patterns of coexpression of tumour-associated antigens CA 19-9, TAG-72, and DU-PAN-2 in human pancreatic cancer. *Int. J. Pancreatol.*, **15,** 97–103.

WARSHAW AL, LEE KH, WOOD WC AND COHEN AM. (1980). Sensitivity and specificity of serum ribonuclease in the diagnosis of pancreatic cancer. *Am. J. Surg.*, **139,** 27–32.

WARSHAW A AND FERNANDEZ-DEL C. (1992). Pancreatic carcinoma. *N. Engl. J. Med.*, **326,** 455–465.

WESLEY A, MANTLE M, MAN D, QURESHI R, FORSTNER G AND FORSTNER J. (1985). Neutral and acidic species of human intestinal mucin. Evidence for different core peptides. *J. Biol. Chem.*, **260,** 7955–7959.

XU M, REAL FX, WELT S, SCHÜSSLER MH, OETTGEN HF AND OLD LJ. (1989). Expression of TAG 72 in normal colon, transitional mucose, and colon cancer. *Int. J. Cancer*, **44,** 985–989.